# Role and therapeutic potential of elabela in renal disease: from molecular mechanisms to clinical applications

**DOI:** 10.1530/EC-25-0410

**Published:** 2025-12-17

**Authors:** Anni Li, Yuxuan Ye, Huimin Cao, Juan Zhang, Yiyuan Zhang, Juan Chen, Min Shi, Hong Zhang

**Affiliations:** Huai’an First People’s Hospital, The Affiliated Huai’an No. 1 People’s Hospital of Nanjing Medical University, Huai’an, China

**Keywords:** elabela, metabolic kidney disease, diabetic nephropathy, chronic kidney disease, acute kidney injury

## Abstract

Elabela (ELA) is a relatively newly identified bioactive micropeptide that functions as the second endogenous ligand for the apelin receptor (APJ). It plays a critical role in diverse physiological processes, including cardiovascular development, blood pressure regulation, and fluid homeostasis. Growing evidence underscores its significance in the pathophysiology of various organ systems, particularly the kidneys. This review aims to comprehensively explore the role of ELA in renal physiology and pathology. We focus on its molecular mechanisms, such as modulating renal hemodynamics, inhibiting fibrosis and inflammation, promoting cellular survival, and its therapeutic potential in acute kidney injury, chronic kidney disease, and hypertensive and diabetic nephropathy. Building upon our research group’s previous work, this article places special emphasis on the role of ELA in renal metabolism and its promising application in the treatment of diabetic kidney disease. By synthesizing recent advancements, we seek to elucidate the connection between ELA and kidney health, assessing its potential as a novel therapeutic target for renal diseases.

## Introduction

A major global public health concern is renal illnesses, which include acute kidney injury (AKI) as well as chronic kidney disease (CKD). A WHO research states that between 8 and 16% of people worldwide have chronic renal disease, and that number is growing (1, 2). In addition to having a significant negative influence on patients’ quality of life, kidney disease has a significant financial cost. The American Kidney Foundation (NKF) estimates that medical expenses related to kidney disease exceed $50 billion annually in the United States alone.

Current treatment methods for kidney diseases primarily include pharmacotherapy, hemodialysis, and kidney transplantation. However, each of these approaches has notable limitations. Pharmacotherapy can only alleviate symptoms and does not provide a cure. While hemodialysis can sustain life, it significantly affects patients’ quality of life and increases the risk of cardiovascular complications with long-term use (3). Although kidney transplantation offers a potential cure, its effectiveness is constrained by the scarcity of donor organs and the risk of rejection (4). Consequently, there is a pressing need for new therapeutic strategies to more effectively prevent and treat kidney diseases, representing a critical focus of contemporary medical research.

Elabela (ELA) is an endogenous peptide hormone, first described in 2013, that functions as a key ligand for the G protein-coupled APJ receptor (also known as the apelin receptor). Its activity can be inhibited by specific APJ receptor antagonists (5). Although elabela shares minimal sequence homology with apelin – about 25% – it retains similarities in the positioning of hydrophobic residues (6). Elabela, though sharing the APJ receptor with apelin, offers unique therapeutic advantages that justify focused research. Structurally distinct and with minimal sequence homology, elabela engages the receptor differently, resulting in unique signaling properties and physiological effects (7). For example, Ho *et al.* showed that the loss of ELABELA rather than apelin caused proteinuria and elevated blood pressure (8). Recent research showed that the APJ receptor, which interacts with elabela, is involved in regulating renal hemodynamics and cell proliferation, highlighting elabela’s key roles in both physiological and pathological states of the kidney (9). All of this evidence points to the common conclusion that elabela and apelin have some different functions, which makes elabela not a redundant ligand but a unique drug worthy of further exploration in the therapeutic field. In particular, elabela’s protective effects during kidney injury and repair suggest its potential as a therapeutic target for CKD as well as AKI. Therefore, a deeper exploration of elabela’s specific mechanisms in renal disease is crucial for developing new therapeutic strategies with greater specificity and fewer side effects.

## Basic properties of elabela

### Genes and structure of elabela

**Figure 1:**
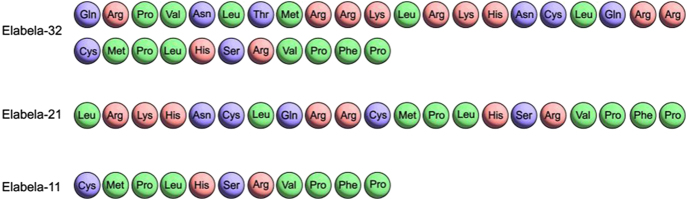
Three functional peptide forms of elabela.

Elabela, or Apela or Toddler, is an important endocrine peptide encoded by a gene located on human chromosome 4q25. This gene produces a precursor peptide composed of 54 amino acids, which is then cleaved by proteases to yield three functional peptide forms: elabela-32 (32 amino acids), elabela-21 (21 amino acids), and elabela-11 (11 amino acids) (shown in Fig. 1). These elabela isoforms can bind to the APJ receptor, with elabela-32 and elabela-21 exhibiting sub-nanomolar and nanomolar affinities for APJ, respectively. This binding affinity is significantly higher compared with that of the shorter elabela-11 peptide (5). The amino acid sequence of the elabela peptide is highly conserved across evolution, indicating its critical biological functions (10).

### Expression distribution of elabela

**Figure 2:**
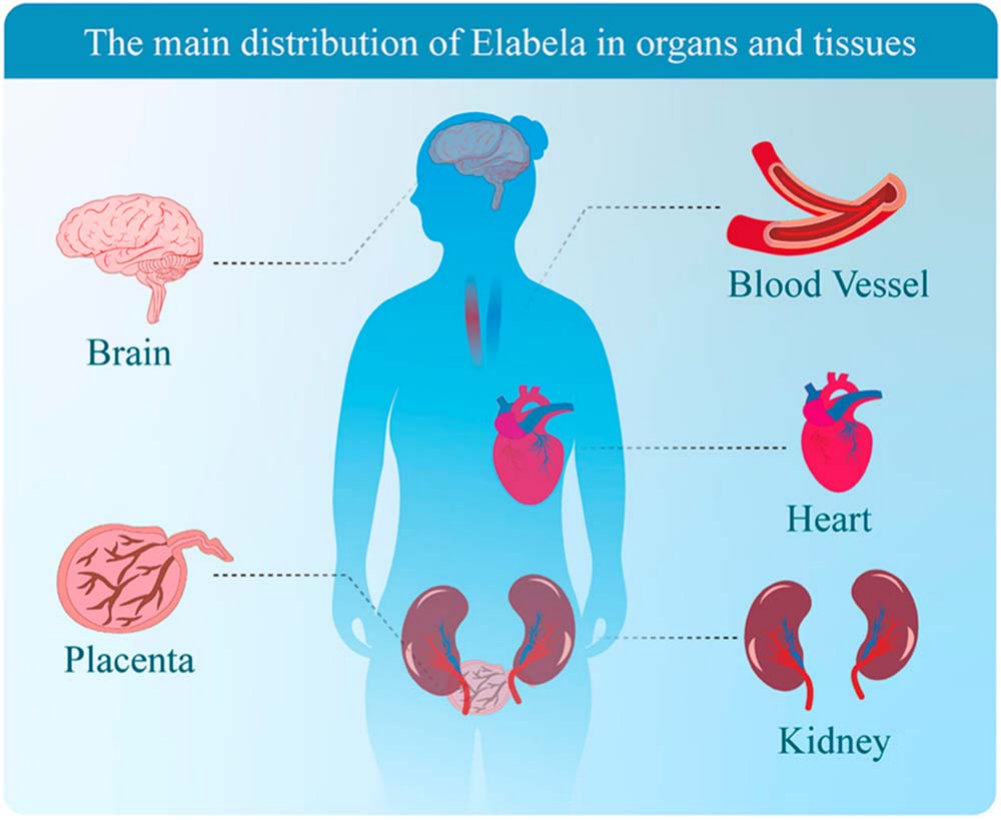
The distribution of organs and tissues in elabela.

The expression and distribution of elabela exhibit notable specificity across different tissues and developmental stages. During embryonic development, elabela is highly expressed in various tissues, particularly in early embryonic stem cells, highlighting its crucial role at critical stages of development (11). Specifically, elabela is abundantly expressed in endodermal cells during early embryonic stages and continues to be present throughout cardiovascular growth, which is vital for the formation of the heart and the vascular system (12). In adults, elabela is predominantly expressed in the cardiovascular system, encompassing cardiac and vascular endothelial cells. Its expression in these tissues is closely associated with cardiovascular protection through the APJ receptor, which helps maintain endothelial function and vascular homeostasis (13, 14). Recent studies have also identified elabela expression in the kidneys; however, the expression of elabela in adult kidneys remains a subject of ongoing investigation and some controversy. While several studies have reported the detection of elabela mRNA and/or protein in renal tissues, suggesting its potential involvement in regulating renal function and blood pressure control (15, 16) (shown in Fig. 2), other reports indicate that its expression in healthy adult kidneys is relatively low or localized to specific compartments (16). This discrepancy could be attributed to differences in detection methodologies, antibody specificities, or the physiological versus pathological states of the studied tissues. Therefore, while Fig. 2 provides a general overview of elabela’s distribution based on the current literature, its expression, particularly in adult organs such as the kidney, may be more nuanced and context dependent (16, 17, 18).

### Main elabela receptor

The coding gene for the G protein-coupled receptor family, which includes the APJ receptor, is APLNR. APJ receptors bind not only to elabela but also to another endogenous ligand, apelin. This receptor is found in many different tissues, including the cardiovascular system, kidneys, lungs, and brain. Studies also highlight that elabela plays a crucial physiological part in the adult cardiovascular system via the APJ receptor. The interaction between elabela and APJ receptors is essential not only for maintaining the steady state of the cardiovascular system but also for providing significant protection under pathological conditions such as stress overload and myocardial injury. In addition, the elabela-APJ axis is crucial in the kidney, where it participates in regulating renal function, controlling blood pressure, and protecting renal microcirculation (15).

## Molecular mechanism

The pleiotropic effects of elabela are mediated through a complex network of signaling pathways, which can be broadly categorized into APJ receptor-dependent and independent mechanisms. Understanding this dichotomy is crucial for appreciating its therapeutic potential in renal diseases.

### Elabela/APJ pathway

Elabela, a key endogenous peptide hormone, is widely recognized for its crucial part in the development as well as maintenance of cardiovascular system functions. Research indicates that elabela (ELA) exerts its biological effects primarily through binding to the APJ receptor (also known as the APJ receptor or ELA receptor), which is intricately involved in various physiological processes within the cardiovascular system (19). Although emerging evidence, particularly in the context of renal studies, suggests that some protective effects of ELA may involve APJ-independent mechanisms, most studies demonstrate that the APJ pathway remains the primary mechanism through which ELA exerts its effects. The activation of these pathways directly mediates the core renal protective effects of ELA, such as promoting cell survival, inhibiting apoptosis, and reducing inflammation and oxidative stress. For example, APJ enhances myocardial contractility as well as glucose uptake in skeletal muscle cells by coupling with Gαq/11 proteins, which activates phospholipase C (PLC) as well as AMPK signaling pathways, While elabela is presumed to share these capabilities due to shared receptor usage, functional data specifically for elabela in these contexts are less extensive (20). The activated APJ receptor then uncouples from G proteins and attracts β-arrestins, starting intracellular signaling cascades that are β-arrestin-dependent and induce receptor endocytosis (21, 22). This phenomenon, known as ‘biased signaling’ or ‘biased agonism’, occurs when different ligands preferentially activate distinct downstream pathways of the same receptor, leading to unique physiological outcomes. These signaling pathways are essential for physiological regulation, including processes such as transcription, cell division, and apoptosis (14).

At the same time, the role of elabela in kidney development and functional regulation has increasingly attracted attention from the scientific community. It was believed that elabela (ELA) was involved in regulating kidney hemodynamics, filtration function, and cell proliferation and differentiation by binding to its specific receptor, APJ (also known as APJ or the ELA receptor). This interaction was thought to be crucial for maintaining kidney function, including filtration and overall water-electrolyte balance (23, 24). In addition, elabela is important for the regulation of renal development and function, with its expression detectable during early renal development, highlighting its key role in renal morphogenesis (25, 26). In CHO cells, Couvineau *et al.* carried out skewed signal transduction studies and *in vitro* pharmacological characterization of apelin as well as elabela/Toddler fragments (27). They evaluated binding affinity, activation of β-arrestin2 recruitment, and suppression of cyclic adenosine monophosphate (cAMP) generation. In order to examine the binding patterns of different endogenous ligands, structural and functional investigations were carried out using site-directed mutagenesis of rat as well as human apelin receptors, as well as alkaline scanning of elabela/Toddler (27). K22P’s alanine scan revealed that its C-terminal region contains the important pharmacophore for receptor binding as well as activation, but none of its cysteine residues were implicated in binding or peptide activity.

The results revealed that residues ASP282 as well as ASP284 in the rat as well as human apelin receptors, respectively, are not involved in elabela/Toddler activity but are crucial for apelin binding as well as activity (27). The differing structural characteristics of elabela/Toddler as well as apelin result in distinct binding patterns of these endogenous ligands to apelin receptors. Recent research has confirmed these findings with greater specificity. Although renal tubules also express APJ receptors, exogenous supplementation of elabela did not significantly improve renal tubular injury, suggesting that elabela’s regulation of renal tubules might not involve APJ receptors (28). Further experiments revealed that elabela overexpression in APJ-knockout cells could mitigate high glucose-induced renal tubular injury. This indicates that elabela may exert its effects on renal tubular cells directly rather than through autocrine or paracrine actions involving the APJ receptor.

#### PI3K/Akt/mTOR signaling pathway

One essential intracellular signaling mechanism that controls cell growth, proliferation, survival, as well as metabolism is the PI3K/Akt/mTOR pathway. Upon binding to the APJ receptor, elabela (ELA) first activates phosphatidylinositol 3-kinase (PI3K). This activation catalyzes the production of phosphatidylinositol 3,4,5-triphosphate (PIP3), a lipid second messenger that recruits and activates protein kinase B (Akt). Activation of Akt initiates a cascade of downstream effects in cells, including the inhibition of pro-apoptotic factors and the promotion of cell survival and growth. Akt also activates mammalian target of rapamycin (mTOR) by directly phosphorylating and inhibiting tuberous sclerosis complex 2 (TSC2) (29). mTOR exists in two complexes: mTORC1 as well as mTORC2, each with distinct functions. While mTORC2 is largely engaged in cytoskeletal reorganization as well as cell survival, mTORC1 principally controls cell growth as well as metabolism.

The activation status of mTORC1 serves as a pivotal switch that intricately links the PI3K/Akt pathway to the regulation of autophagy (19). While the PI3K/Akt/mTOR axis typically inhibits autophagy, elabela-APJ signaling can fine-tune this process in a context-dependent manner to promote cellular homeostasis. For instance, under conditions of cellular stress such as nutrient deprivation or in specific pathological contexts such as renal cancer, ELA can paradoxically enhance autophagic flux. This is achieved by modulating mTORC1 activity and through parallel mechanisms, such as activation of the energy-sensing kinase AMPK, a potent inducer of autophagy that inhibits mTORC1 (30). Furthermore, Chen *et al.* demonstrated that ELA can reverse the downregulation of the transcription factor EB (TFEB), a master regulator of lysosomal biogenesis, thereby restoring autophagic flux and improving lysosomal function (31). This coordinated action – promoting survival signals via PI3K/Akt while simultaneously facilitating the clearance of damaged organelles and proteins via autophagy under stress – highlights a sophisticated mechanism through which the ELA-APJ axis confers cytoprotection (32, 33).

**Figure 3:**
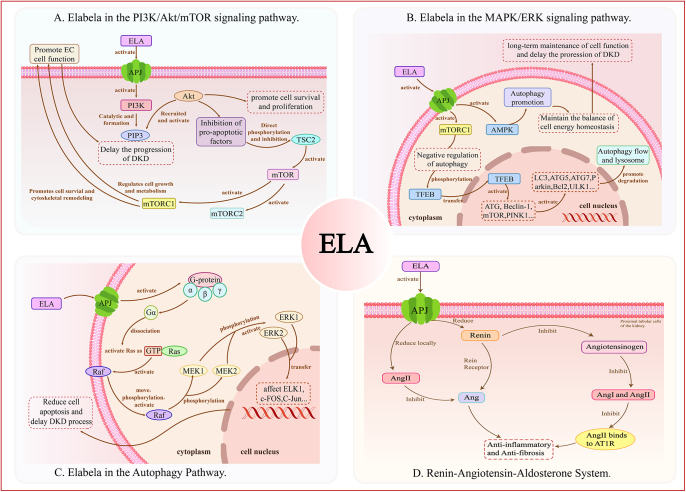
The main mechanism of elabela. (A) The role of elabela in the PI3K/Akt/mTOR signaling pathway. ELA activates the APJ receptor to trigger the PI3K/Akt/mTOR pathway. This cascade promotes endothelial cell (EC) function, cell survival and proliferation, and regulates cell growth and metabolism, while inhibiting pro-apoptotic factors to delay DKD progression. (B) The role of elabela in the MAPK/ERK signaling pathway. ELA-APJ activation drives the MAPK/ERK pathway via G-protein and Ras/Raf/MEK/ERK signaling. Phosphorylated ERK transfers to the nucleus to affect transcription factors (e.g., ELK1), reducing cell apoptosis and retarding DKD progression. (C) The role of elabela in the autophagy pathway. ELA modulates autophagy via APJ-mediated regulation of mTORC1 and AMPK. It promotes autophagy (via TFEB and autophagy-related factors) and maintains energy homeostasis, supporting long-term cell function. (D) The role of elabela in the renin-angiotensin system. ELA acts on APJ to reduce local AngII and inhibit the renin-angiotensin system. This suppresses AngII-AT1R binding, exerting anti-inflammatory and anti-fibrotic effects to protect renal tubular cells. PI3K: phosphatidylinositol 3-kinase; PIP3: phosphatidylinositol (3,4,5)-trisphosphate; AKT: serine/threonine kinase; mTORC1: mechanistic target of rapamycin complex 1; mTORC2: mechanistic target of rapamycin complex 2; mTOR: mechanistic target of rapamycin; TSC2: tuberous sclerosis complex 2; AMPK: AMP-activated protein kinase; TFEB: transcription factor EB; LC3: microtubule-associated protein 1A/1B-light chain 3; ATG5: autophagy-related 5; ATG7: autophagy-related 7; ATG: autophagy-related protein; Bcl2: B-cell lymphoma 2; ULK1: Unc-51-like autophagy activating kinase 1; PINK1: PTEN-induced kinase 1; Raf: rapidly accelerated fibrosarcoma; MEK1: mitogen-activated protein kinase kinase 1; MEK2: mitogen-activated protein kinase kinase 2; ERK1: extracellular signal-regulated kinase 1; ERK2: extracellular signal-regulated kinase 2; ELK1: ETS-like protein 1; C-FOS: FBJ osteosarcoma oncogene; C-Jun: jun proto-oncogene; AT1R: angiotensin II receptor type 1; Ang II: angiotensin II.

The PI3K/Akt/mTOR signaling pathway, activated by the ELA-APJ axis, not only promotes cell proliferation and survival but also plays a vital part in embryonic growth, cardiovascular function maintenance, as well as metabolic homeostasis (19). Further validation by Wang *et al.* using the PI3K inhibitor wortmannin demonstrated the beneficial effects of ELA on endothelial cell (EC) function as well as APJ receptor expression were hindered by suppression of PI3K/Akt signaling. This suggests that ELA may modulate EC function through the ELA-APJ axis and the PI3K/Akt signaling pathway (34). Other researchers’ further *in vitro* and *in vivo* experiments have verified that the PI3K/Akt signaling pathway mediates ELA’s anti-apoptotic and preventive effects (35) (shown in Fig. 3A).

#### MAPK/ERK signaling pathway

Elabela promotes the extracellular signal-regulated kinase (ERK) signaling pathway, which is essential for cell survival, differentiation, as well as proliferation, via binding to the APJ receptor (33). In the renal context, ERK pathway activation is crucial for the repair and regeneration of renal tubular epithelial cells after AKI. It accelerates the structural and functional recovery of renal tissue by promoting cell cycle progression and migration. Specifically, elabela initiates signaling by binding to its specific G protein-coupled receptor, APJ, triggering a conformational change in the receptor and activating the associated G proteins. The activated Gα subunit dissociates and activates the small GTPase Ras. Activated Ras then promotes the activation of Raf kinases (A-Raf, B-Raf, and C-Raf), which phosphorylate MEK kinases (MEK1 as well as MEK2), leading to the phosphorylation as well as activation of ERK kinases (ERK1 as well as ERK2). Activated ERK translocates to the nucleus, regulating various transcription factors such as ELK1, c-Fos, and c-Jun, thereby affecting genes related to cell proliferation, differentiation, and survival (shown in Fig. 3B and C). In the context of vascular repair, ERK activation promotes endothelial cell cycle progression, proliferation, and migration. Furthermore, ERK signaling has also been implicated in the regenerative processes of renal tubular epithelial cells after AKI.

#### Antagonism of the renin-angiotensin system (RAAS)

The elabela (ELA)-APJ system has emerged as a significant endogenous regulatory pathway that interacts with the renin-angiotensin-aldosterone system (RAAS) through multiple mechanisms to maintain cardiovascular and renal homeostasis. Extensive research demonstrates that ELA binding to the APJ receptor directly suppresses key components of the RAAS cascade, particularly in renal tissues. Experimental evidence reveals that ELA significantly inhibits the expression of the (pro)renin receptor (PRR), soluble PRR, and renin itself within the renal collecting duct system, thereby reducing angiotensin II (Ang II) production at its source. This upstream suppression of RAAS activation contributes to blood pressure reduction and renal protection, establishing the ELA-APJ axis as a crucial counter-regulatory mechanism (18). This establishes the ELA-APJ axis as a counter-regulatory system to the classical RAAS. Studies have shown that ELA-21 treatment alleviates Ang II-induced inflammation, oxidative stress, and VSMC proliferation, with these adverse effects intensifying when ELA expression is suppressed (36). Furthermore, the key advantage of ELA over apelin-13 lies in its remarkable resistance to degradation by RAAS enzymes. This unique metabolic stability endows it with superior pharmacokinetic properties, enabling sustained modulation of RAAS activity (shown in Fig. 3D). Combined with its multi-level inhibitory effects on RAAS, the ELA-APJ axis demonstrates significant potential for treating hypertension and related cardiorenal diseases.

### Elabela/non-APJ pathway

In addition to the APJ (apelin receptor, APLNR), apelin and elabela (ELA) may also exert their functions through non-APLNR-dependent pathways. It has been observed that exogenous ELA and apelin can significantly enhance the invasiveness and differentiation of trophoblasts, and these effects are not affected by APLNR antagonists or APLNR knockdown in some experiments, suggesting the existence of non-dependent mechanisms (7). Similarly, while ELA’s effect in alleviating diabetic cardiomyopathy is partially dependent on the NF-κB pathway activated by APLNR, its ameliorative effects on diabetic cardiomyopathy are not completely eliminated when APJ is knocked out in endothelial cells (37). These findings imply that there may be some undiscovered receptors that can bind with apelin/ELA in addition to APLNR.

## Elabela and renal disease

Studies have shown that administering apelin or elabela (ELA), either into the lateral ventricle or intravenously, can significantly increase urine output in animals, which is closely linked to kidney function (38). This suggests that ELA may regulate water and salt balance by inhibiting renal tubule reabsorption of water (e.g., by regulating aquaporin) or by affecting glomerular filtration rate, which is an important manifestation of its maintenance of endogenous homeostasis and protection of renal function. In cases of AKI as well as hypertensive nephropathy, the protective effects of elabela are primarily seen in its ability to regulate renal blood flow, inhibit inflammatory responses, and promote renal repair. In contrast, in CKD, elabela’s role is more focused on inhibiting renal fibrosis and managing persistent inflammatory responses. This difference suggests that elabela may exert its effects through various mechanisms depending on the pathological state, offering a multi-dimensional approach to treatment.

### Acute kidney injury (AKI)

AKI, characterized by a rapid decline in renal function due to ischemia, toxicity, or inflammation, represents a significant clinical challenge (39, 40). In recent years, there has been increasing attention about the role of the endogenous peptide hormone elabela (ELA) in AKI, particularly its potential protective effects. ELA is primarily secreted and expressed in normal renal tubular epithelial cells, while its expression levels change significantly in the context of AKI. For instance, mRNA as well as protein levels of ELA in the kidney were decreased during the early post-injury phase in an ischemia-reperfusion (I/R)-induced AKI model, suggesting that depletion of endogenous ELA may exacerbate the sensitivity of the kidney to acute injury. Therefore, exogenous ELA supplementation is not only a replacement therapy but also a precise intervention strategy targeting the lack of endogenous protective substances (41). In another study, the ELA-32 analog (the N-terminal cysteine is substituted (AE11C)) inhibited I/R injury-induced renal fibrosis, inflammation, apoptosis, as well as DNA damage. This treatment resulted in a reduction in tubular lesions and renal dysfunction (28). Later, Chen *et al.* utilized siRNA to knock down APJ in NRK-52E cells (a rat renal tubular epithelial cell line) subjected to ischemia/reperfusion (I/R) conditions. This intervention dramatically increased cell viability and reduced DNA damage and inflammatory responses, indicating that elabela’s protective actions in I/R may involve additional, as yet undiscovered, receptors and are not dependent on APJ.

In addition, the protection of the ELA peptide in AKI has been explored by exogenous administration. The findings revealed that ELA treatment improved renal function in an AKI model. Mechanistically, this protection is achieved by activating the PI3K/Akt/mTOR pathway to suppress tubular cell apoptosis, inhibiting the NLRP3 inflammasome to reduce inflammatory responses, and mitigating oxidative stress via the AMPK/NOX4 pathway (15). These results suggest that ELA may be involved in kidney protection and repair through a variety of mechanisms, including but not limited to reducing inflammatory responses, inhibiting apoptosis, and promoting kidney cell regeneration.

In summary, ELA counters AKI through a coordinated mechanism involving the suppression of apoptosis, inflammation, and oxidative stress. While the translational promise of ELA is significant, next steps must delineate its context-dependent signaling and optimize the delivery of ELA-based therapeutics for clinical application.

### Chronic kidney disease (CKD)

A gradual loss of renal function as well as continuous damage to renal tissues is a hallmark of CKD, a worldwide health concern. Key pathophysiological processes in CKD progression include renal fibrosis and persistent inflammation (42). Recently, the role of the endogenous peptide hormone elabela (ELA) in regulating these processes has garnered significant attention.

A central mechanism involves the attenuation of renal fibrosis. Research indicates that ELA can inhibit the profibrotic transforming growth factor-β (TGF-β) signaling pathway, a major driver of extracellular matrix (ECM) accumulation, thereby directly countering the fibrotic process (43). Beyond its antifibrotic actions, ELA modulates the chronic inflammatory milieu of CKD. It is suggested to reduce the production of pro-inflammatory cytokines, such as TNF-α and IL-1β, potentially through APJ receptor-mediated activation of anti-inflammatory signaling pathways, which helps to dampen the persistent inflammatory state that exacerbates kidney damage (44).

Clinically, the relevance of these mechanisms is supported by the correlation between serum ELA levels and disease severity. Studies consistently show that circulating ELA levels decrease as CKD advances and are inversely correlated with markers of renal dysfunction and hypertension (26, 45). These correlations position ELA as a potential biomarker that inversely reflects the severity of CKD, encompassing its hallmark features of declining filtration function, rising blood pressure, and the development of renal anemia.

These clinical associations, while compelling, underscore the need to move beyond correlation and elucidate the precise cause-and-effect mechanisms by which ELA may influence renal fibrosis and the chronic inflammatory milieu that drives CKD progression.

#### Hypertensive nephropathy

Hypertensive nephropathy and malignant arteriolosclerosis are characterized by hypertension and renal failure. In these conditions, renal tubular damage often occurs before glomerular damage. Clinical manifestations include nocturia, a decline in urine concentration, a lighter urine color, mild proteinuria, and possible microscopic hematuria and casts. These patients frequently have complications affecting other target organs due to hypertension (46).

Elabela (ELA) plays a critical role in counteracting both systemic hypertension and the resultant renal injury through distinct yet interconnected molecular pathways. Serum ELA levels are inversely correlated with blood pressure severity and the degree of renal impairment. For instance, hypertensive patients have lower serum elabela levels than healthy controls, which decrease with higher blood pressure. Elabela levels are negatively correlated with blood pressure and kidney damage markers but positively correlated with kidney function. Malignant hypertension patients show more severe kidney damage and even lower elabela levels (44, 47, 48).

The regulatory capacity of ELA on vascular biology, initially described in embryonic angiogenesis where it guides angioblast migration via the APJ receptor (23), is repurposed in the diseased adult kidney to promote vascular stability and repair. Mechanistically, ELA regulates systemic blood pressure primarily via APJ receptor-dependent pathways. This includes inducing vasodilation through nitric oxide production and, more significantly, suppressing the intrarenal (pro)renin receptor (PRR) and renin activity, thereby functioning as an endogenous antagonist of the renin-angiotensin system (RAS) (17, 18, 49). Beyond hemodynamic regulation, ELA directly mitigates hypertensive renal damage. It counteracts renal fibrosis by downregulating key profibrotic markers, including collagen and α-SMA (43). Furthermore, ELA protects the kidney by inhibiting the NADPH oxidase/ROS/NLRP3 pathway, thereby reducing oxidative stress and inflammatory activation (32). Notably, some of these renoprotective effects, such as inhibition of the NADPH oxidase/ROS/NLRP3 pathway and attenuation of fibrosis, have been observed even in the absence of the APJ receptor, suggesting the involvement of additional, noncanonical mechanisms (18, 32).

In summary, elabela demonstrates significant potential in regulating blood pressure and alleviating kidney damage in hypertensive nephropathy.

#### Diabetic kidney disease (DKD)

A typical side effect of diabetes is diabetic nephropathy, which features loss of kidney function as well as increased urine protein excretion. This condition manifests as glomerular hypertrophy, impaired filtration, and renal fibrosis (50). In 1936, Kimmelstiel and Wilson described diffuse and nodular glomerulosclerosis, which shifted research focus primarily to the glomerulus, aiding in the understanding of diabetic nephropathy pathogenesis (51). However, subsequent findings by Richard revealed that some patients with advanced diabetic nephropathy exhibited neither significant glomerular lesions nor proteinuria, and their kidney function remained within traditional renal disease indicators (52). Consequently, research on diabetic nephropathy shifted toward the renal tubules. ELA, a peptide secreted by renal tubules, has drawn considerable attention in the field of DKD research. Early studies also showed that another APJ ligand, apelin, is associated with glucose uptake. Cedric *et al.* found that acute intravenous injection of apelin in fed mice resulted in a potent hypoglycemic effect, linked to higher glucose utilization in skeletal muscle as well as adipose tissue. It was shown using pharmacological, genetic, and *in vivo* techniques that apelin promotes glucose absorption in the soleus muscle via Akt, AMP-activated protein kinase, and endothelial nitric oxide synthase. Furthermore, apelin was shown to improve glucose utilization and restore glucose tolerance in obese as well as insulin-resistant rats (53).

Later research revealed a strong correlation between the onset and advancement of diabetic nephropathy and the expression levels of elabela, another APJ ligand. In high-glucose environments, elabela expression is upregulated, leading to a reduced glomerular filtration rate as well as the onset of proteinuria (8, 54, 55). Further investigations revealed that elabela in renal tubular cells does not act through the previously identified APJ receptor but instead influences tubular reabsorption by regulating the ubiquitin-mediated degradation of the epithelial sodium channel (ENaC) (6). These findings warrant additional basic research for confirmation. As a critical regulatory factor in diabetic glomerular injury, previous studies have demonstrated that podocyte injury and a reduction in podocyte number are closely linked to the development of proteinuria in DKD (56). In diabetic conditions, ELA exerts protection via distinct mechanisms in different renal cell types. In podocytes, ELA activation of the PI3K/AKT/mTOR pathway attenuates apoptosis and restores the expression of slit diaphragm proteins such as synaptopodin and podocin (57).

In the specific context of DKD, clinical research demonstrates that serum elabela levels are particularly low and correlate negatively with disease severity (serum creatinine and ACR). This suggests that the hyperglycemic and profibrotic milieu of diabetes may specifically suppress ELA expression or accelerate its consumption, which could contribute to the loss of its putative renoprotective effects and thereby exacerbate disease progression. In addition, animal experiments conducted by Zhang Hong *et al.* confirmed that exogenous elabela peptide counteracts diabetes-related kidney injury and fibrosis, with elabela treatment significantly inhibiting fibrosis-related proteins in the kidneys of db/db mice (58). These results suggest that elabela may serve as an important biomarker for predicting diabetic kidney injury. A study of 100 diabetic patients and 50 controls found that serum ELA levels decreased progressively with worsening albuminuria severity. ELA levels correlated negatively with uremia, retinopathy, hyperglycemia, and LDL-C but positively with eGFR (59).

Although clinical studies have suggested an association between decreased ELA levels and DKD, its potential as a specific biomarker still requires cautious evaluation. Since changes in ELA levels may be commonly present in various diabetic complications, such as nephropathy, retinopathy, and peripheral neuropathy, its specificity for DKD is likely limited. Furthermore, existing research is predominantly cross-sectional in design, which imposes limitations on establishing causality, while issues such as insufficient sample size may also affect the generalizability of the findings. Therefore, more longitudinal studies are needed to validate whether ELA can be established as a reliable prognostic indicator or therapeutic target.

## Discussion

The burgeoning research on elabela underscores its significant promise as a novel therapeutic target for a spectrum of renal diseases. Its endogenous nature suggests a favorable biocompatibility profile, potentially minimizing adverse reactions compared to conventional therapies. The diverse mechanisms of action of elabela, encompassing both APJ-dependent and independent pathways, provide a multifaceted approach to treating kidney injury by targeting hemodynamics, fibrosis, inflammation, and autophagy.

Our review highlights that beyond its local renal actions, elabela acts as a systemic endocrine regulator. Its therapeutic potential is deeply rooted in its ability to fine-tune critical endocrine functions, including the antagonism of the renin-angiotensin system, the regulation of sodium handling, and the modulation of systemic metabolic pathways, all of which are frequently disrupted in renal patients.

Several strategies are being explored to translate this potential into clinical reality. First, overcoming the inherent limitation of peptide degradation and short half-life is paramount. For example, ELA11’s therapeutic use was restricted by further pharmacological investigations that revealed its low plasma stability and comparatively weak binding to APJ (relative to the longer isoforms ELA-32 and ELA-21) (60). Structure–activity relationship studies have led to the development of engineered analogs, such as those modified with polyethylene glycol (PEG) or palmitic acid (Pal), which demonstrate enhanced plasma stability and sustained receptor binding, resulting in superior renal protection in AKI models compared to the native peptide (61). Second, the design of Fc-elabela fusion proteins (where elabela is fused with the crystallizable fragment of immunoglobulin to extend its plasma half-life) represents a breakthrough, significantly prolonging its circulation time and proving highly effective in ameliorating inflammation and apoptosis in models of LPS-induced AKI and renal ischemia-reperfusion injury (62, 63). Apart from these engineered approaches, the discovery of a naturally occurring, more stable metabolite, ELA-16, suggests that elabela itself may serve as an excellent template for developing stable drugs targeting the apelin receptor (60). Third, the potential of elabela extends beyond fibrotic and ischemic injuries. In renal cell carcinoma, elabela has been shown to inhibit tumor growth and angiogenesis by modulating mTORC1 signaling, and synergistic effects with anti-cancer drugs such as sunitinib have been observed, opening a new avenue for oncotherapeutic applications (30).

Despite the optimistic outlook, several challenges must be addressed to advance elabela toward clinical application. Key unanswered questions persist, including the definitive cellular source of renal elabela in adults, the molecular identity of its APJ-independent targets, and whether its role shifts from protective to harmful in advanced CKD. The precise mechanisms of action across various renal pathologies remain incompletely understood, particularly regarding APJ-independent signaling and potential alternative receptors. Furthermore, developing innovative delivery strategies is essential to achieve renal-specific targeting while minimizing systemic effects.

The potential for off-target cardiovascular effects due to systemic APJ activation by elabela warrants careful investigation. Although elabela demonstrates cardioprotective effects in physiological conditions, its systemic administration at therapeutic doses may induce complex hemodynamic responses. Studies indicate that elabela-APJ signaling exhibits dose-dependent and context-specific characteristics, where excessive activation could potentially lead to adverse effects, including hyperthyroidism or hypertension, in susceptible individuals (14, 64, 65). Another study found that microinjection of ELA-21 into the PVN increases sympathetic nerve activity and blood pressure (66). Clinical studies have also shown elevated elabela levels in STEMI patients (67). The therapeutic application of elabela presents a double-edged sword. Systemic administration, particularly at high doses, may induce excessive blood pressure promotion, posing risks for patients with pre-existing hypertension or hemodynamic instability. While its short half-life potentially reduces long-term exposure risks, this pharmacokinetic characteristic simultaneously challenges maintaining stable therapeutic concentrations. Therefore, developing structurally modified elabela analogs with optimized pharmacokinetics, alongside conducting thorough experimental evaluations to determine the therapeutic window and establish its risk–benefit profile, constitutes a critical research direction. Furthermore, the potential for off-target cardiovascular effects due to systemic APJ receptor activation warrants careful investigation. While the endogenous elabela-APJ axis is cardioprotective, pharmacological administration must be optimized to avoid unintended hemodynamic consequences. Future efforts should focus on developing renal-targeted delivery systems or biased ligands (biased ligands preferentially activate specific downstream pathways while minimizing activation of alternative pathways in GPCR signaling) to maximize cardiorenal benefits and minimize systemic risks (27). The development of highly selective and potent agonists remains a critical hurdle for achieving optimal therapeutic outcomes with minimal off-target effects. Furthermore, the interplay between elabela and existing therapies, such as RAS inhibitors (ACEIs/ARBs) or SGLT2 inhibitors, warrants thorough investigation to explore potential synergistic benefits. Finally, while genetic studies suggest that modulating elabela expression could be a powerful strategy (43, 68), the safety and efficacy of long-term elabela supplementation or gene therapy require validation through large-scale preclinical and clinical trials.

In conclusion, while the path forward involves overcoming significant translational challenges, the unique attributes of the elabela system offer a fresh and compelling perspective for innovative kidney disease treatment. Continued research efforts focused on elucidating its detailed mechanisms, optimizing drug design, and conducting rigorous clinical trials are essential to fully realize the therapeutic potential of this intriguing peptide hormone.

## Declaration of interest

The authors declare that they have no known competing financial interests or personal relationships that could have appeared to influence the work reported in this paper.

## Funding

This work was supported by grants from the Science and Technology Department of Jiangsu Provincial (BE2023745) and Health Commission of Jiangsu Province (H2023137) to HZ.

## Author contribution statement

Li Anni was responsible for writing the original draft. Yuxuan Ye, Huimin Cao, Juan Zhang, Yiyuan Zhang, Juan Chen, Min Shi, and Hong Zhang were responsible for writing review and editing.
